# The complete plastid genome and phylogenetic analysis of *Gracilaria chilensis*

**DOI:** 10.1080/23802359.2018.1431070

**Published:** 2020-03-02

**Authors:** Guoliang Wang, Na Liu, Yue Li, Lei Zhang, Maria Dyah Nur Meinita, Weizhou Chen, Tao Liu, Shan Chi

**Affiliations:** a College of Marine Life Sciences, Ocean University of China, Qingdao, China;; b CAS Key Laboratory of Genome Sciences and Information, Beijing Institute of Genomics, Chinese Academy of Sciences, Beijing, China;; c University of Chinese Academy of Sciences, Beijing, China;; d Faculty of Fisheries and Marine Science, Jenderal Soedirman University, Purwokerto, Indonesia;; e Marine Biology Institute, Shantou University, Shantou, China;; f Qingdao Haida BlueTek Biotechnology Co., Ltd, Qingdao, China

**Keywords:** *Gracilaria chilensis*, complete plastid genome, Gracilariaceae, phylogenetic analysis

## Abstract

*Gracilaria chilensis* is an economically important species of macroalgae. The plastid genome sequence of *G. chilensis* is 185,640 bp with a GC content of 29.34%. A total of 236 genes were determined, containing 203 protein-encoding genes, three rRNA genes, 30 tRNA genes, and one intron (with intronic ORF) inserted into the *trnM* gene. The gene content and structure of Gracilariaceae species were relatively well conserved. The phylogenetic analysis, based on the red algal plastid genomes, suggested that *G. chilensis* had a closer relationship with *Gracilaria tenuistipitata* var. *liui* in *Gracilaria*.

*Gracilaria chilensis* is an economically important macroalga in the family Gracilariaceae. This family includes ∼230 species in seven genera (Lyra et al. 2015). Currently, over 150 species of *Gracilaria* have been described. *G. chilensis*, distinguished by Bird et al. (1986), is a natural marine food with high fibre, high protein, low fat, low calories, and is rich in vitamins and minerals. Moreover, it is the most economically important alga, mainly used as a high-quality raw material for extracting agar and algin (Meinita et al. 2017). Besides, it is believed that *G. chilensis* can be used for bioremediation of ocean acidification, by absorbing excess CO_2_ from seawater (Gao and McKinley 1994). Both of these economic and environmental advantages can be achieved by integrating the cultivation of algae with fish farming.

In this study, we provide the red algal plastid genome by next-generation sequencing methods. The specimen was collected from Rongcheng, Shandong Province of China (37°09′52″N, 122°28′53″E), and stored in the Culture Collection of Seaweed at the Ocean University of China (sample accession number: 2016070050). Total DNA was extracted via the modified CTAB method (Doyle and Doyle 1990). The genome data of *G. chilensis* was obtained using an Illumina HiSeq system with paired-end, 150 bp sequencing. The complete plastid genome, using *Gracilaria salicornia* (NC_023785) as reference, was annotated with Geneious R10.1.3. The plastid genome map of *G. chilensis* was produced using OGDRAW software.

The complete plastid genome of *G. chilensis* was mapped as a circular molecule of 185,640 bp with 29.34% GC content (GenBank accession number MF401963), including 203 protein-encoding genes, three rRNA genes, 30 tRNA genes, and one intron interrupting the *trnM* gene. The nucleotide composition was 35.53% A, 14.66% C, 14.69% G, and 35.13% T. The length of the coding region was 145,521 bp, corresponding to 78.4% of the total length. The plastid genome of *G. chilensis* was compact, with 10 pairs of overlapping genes found with overlap lengths of 1–95 bp (*carA*-*ycf53*, *psbD*-*psbC*, *ycf29*-*trnH*, *trnT*-*ilvB*, *rpl24*-*rpl14*, *rpl14*-*rps17*, *rps17*-*rpl29*, *rpl23*-*rpl4*, *rps18*-*rpl33*, and *atpF*-*atpD*). Synteny analysis of species published in the NCBI sequence database revealed that the size of the plastid genomes of Gracilariaceae was 180–185 kb, with small differences in GC content (average GC content, 28.41%). The gene numbers and structures were largely similar among Gracilariaceae species; their plastid genomes were relatively well conserved, with no gene rearrangement phenomena.

Phylogenetic analysis was conducted using 85 shared plastid protein sequences from 17 red algal plastid genomes, publicly available from the GenBank dataset, and using *Cyanidioschyzon merolae* as an outgroup. Our results indicated that all red algal taxa were clearly separated into different groups, according to their original class (Figure 1). The Florideophyceae species formed a large branch, in which five species from the Gracilariales order were grouped as a sub-branch, in which *G. chilensis* showed a closer relationship with *Gracilaria tenuistipitata* var*. liui* in *Gracilaria.* This study also facilitated a novel interpretation of the evolutionary process from ancient unicellular algae to large multicellular complex algae.

**Figure 1.**
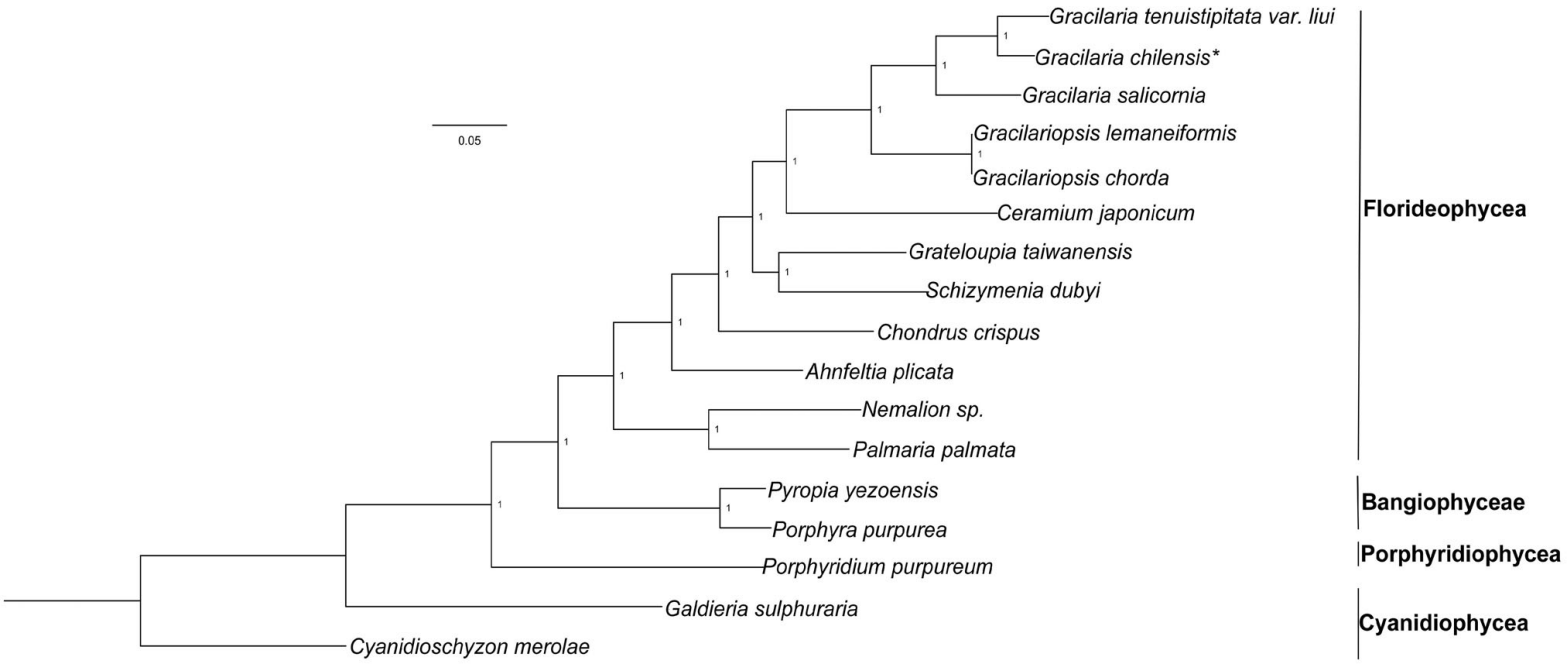
Phylogenetic analysis was performed using a Bayesian method, based on the plastid genomes of red algae as shown below: *Cyanidioschyzon merolae* (NC_004799), *Galdieria sulphuraria* (KJ700459), *Porphyridium purpureum* (NC_023133), *Pyropia yezoensis* (KC517072), *Porphyra purpurea* (U38804), *Palmaria palmata* (NC_031147), *Nemalion sp.* (LT622871), *Ahnfeltia plicata* (NC_031145), *Chondrus crispus* (NC_020795), *Ceramium japonicum* (NC_031174), *Grateloupia taiwanensis* (KC894740), *Schizymenia dubyi* (NC_031169), *Gracilariopsis chorda* (NC_031149), *Gracilariopsis lemaneiformis* (KP330491), *Gracilaria salicornia* (NC_023785), *Gracilaria tenuistipitata var. liui* (AY673996), and *Gracilaria chilensis* (MF401963). Asterisk indicates newly sequenced species in this study.
